# Increased use of Dermoscopy in Primary Healthcare Following the Implementation of Teledermatology in Southeast Sweden: A Retrospective Cohort Study of 2,137 Patients

**DOI:** 10.2340/actadv.v104.40890

**Published:** 2024-11-13

**Authors:** Christofer SAHIN, Mattias B. CARLSSON, Fredrik Munir EHRLINGTON, Emanuela MICU, Magnus FALK

**Affiliations:** 1 Department of Dermatology and Venereology in Östergötland, and Department of Biomedical and Clinical Sciences, Linköping University, Linköping; 2 Department of Health, Medicine and Caring Sciences, Linköping University, Linköping; 3 Department of Dermatology and Venereology in Östergötland, Vrinnevi Hospital, Norrköping, Sweden

**Keywords:** carcinoma, basal cell carcinoma, squamous cell carcinoma, dermoscopy, melanoma, skin neoplasms, telemedicine

## Abstract

In the last 5 decades there has been a steady increase in skin cancer incidence globally. As patients wait for treatment before or after referral, the prognosis for those with melanoma worsens. Teledermatology was introduced to help reduce waiting times. The objective of this study was to investigate how the introduction of teledermatology affected management of skin tumours, from primary care physicians to dermatologists. A retrospective cohort study was performed 1 year before and 1 year after introduction of teledermatology in Östergötland County, Sweden. Patients were included from 3 primary healthcare centres by 3 independent observers. A total of 2,139 patients were included in the study. The 2 cohorts were well matched. At 2 of the 3 primary healthcare centres there was a significant increase in the use of dermoscopy, and almost 66% of all referrals were teledermatological in the year following its introduction. There was a trend towards higher diagnostic accuracy in the post-teledermatology cohort. No apparent effect on melanoma referral times was observed. The results of this study confirm previous findings showing the value of teledermatology as well as a novel finding of an increase in dermoscopy use in primary healthcare settings.

## SIGNIFICANCE

More and more people worldwide suffer from skin cancer. Waiting to see a doctor could worsen the prognosis. Teledermatology, the process where images of skin lesions are sent from primary healthcare to a dermatologist, speeds up the referral process and makes it easier for dermatologists to prioritize referrals. The aim of this study was to look at how introducing teledermatology in Southeast Sweden affected this process. The results show that dermoscopy use increased among primary healthcare physicians, which could increase their diagnostic capabilities over the long term, helping ensure that more patients who need urgent care receive it.

In the last 5 decades there has been a steady increase in the incidence of skin cancers such as melanoma, basal cell carcinoma (BCC), and squamous cell carcinoma (SCC) in Sweden, in both men and women (1). This increase in incidence has also been seen globally, mostly in populations with European heritage (2, 3).

Melanoma is the leading cause of mortality due to skin cancer (4). Mortality is strongly correlated with several factors, including the thickness of the melanoma and whether it is ulcerated, where a thicker ulcerated melanoma has a worse prognosis (5). The longer a melanoma remains untreated, the more advanced the tumour might become, and the worse the prognosis, which is why early diagnosis is important in increasing survival (6). Naturally, increased skin cancer incidence also means more patients in circulation, which means a higher burden on the whole healthcare system with the resulting risk of longer waiting times for dermatologist consultations both before and after referral. There is an urgent need to rapidly adjust the system to the steep increase in skin cancer incidence.

One way of improving the management of skin lesions – such as finding potentially fatal melanomas early as well as avoiding unnecessary referrals and excisions – is by strengthening communication between primary care physicians (PCP) and dermatologists through teledermatology (7, 8). Teledermatology is a means of digitally communicating with a dermatologist and is normally done in 1 of 2 ways: (*i)* “Store and forward” occurs when digital images, including, if available, a close up using a dermatoscope add-on on the camera for greater detail, taken by a PCP, and sent alongside a digital referral/consultation, where they are reviewed by a dermatologist who can either reply with a recommendation, accept the referral and see the patient themselves, or send the patient straight to surgery for removal. (*ii)* Video consultations, where dermatologist, referrer, and patient all meet and can have a live interaction (6, 9).

**Fig. 1:**
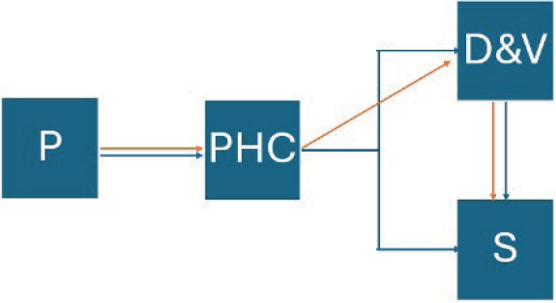
Patient flowchart. P: patient; PHC: primary healthcare centre; D & V: dermatology and venereology clinic; S: surgical clinic. Blue arrows: pre-teledermatology; orange arrows: post-teledermatology.

The Swedish health system provides universal health coverage for all residents, regardless of nationality. Region Östergötland, 1 of 21 counties in Sweden, has a responsibility for providing health services to its inhabitants, with primary healthcare serving as the first line of care, where the initial assessment of most medical conditions is made (**Fig. 1**). Before the introduction of teledermatology a patient presented at a primary healthcare centre (PHC) and a PCP then referred the patient, without images, either directly to a surgical clinic or to the dermatology clinic for assessment, which, in turn, might refer the patient onwards to surgery. Since teledermatology was introduced the PCP refers the patient with images, macroscopic and dermoscopic (henceforth included in the term teledermatology), of the suspected lesion making it easier for the dermatologist to prioritize incoming referrals according to severity and directly refer the patient onwards to surgery if deemed necessary without a personal visit to the dermatology clinic, eliminating unnecessary wait.

Previous studies have shown that, in general, teledermatology may be an effective, easy-to-use and cost-effective tool, improving access to care and patient satisfaction and ensuring a high degree of diagnostic accuracy when it comes to skin tumours – provided it is implemented properly (10). Some reported pitfalls are a lack of information in the referral, poor image quality, inability to palpate/investigate the lesion in person, and an inability to perform complete physical examination (9, 11). How introducing teledermatology itself has affected the use of dermoscopy in the primary healthcare setting has not, to the best of our knowledge, been studied previously. However, studies do show that there is a wide variance in agreement when it comes to diagnosis between PCPs and dermatologists, as well as in the access and use of dermatoscopes in the primary healthcare setting (12, 13).

Starting in 2016, teledermatology was introduced at a selected number of PHCs in Östergötland County, in south-east Sweden, before being fully implemented. Based on previous epidemiologic studies, Östergötland County has been shown to be a feasible representative for Sweden at large when it comes to incidence and mortality rates of skin cancer as well as in clinical management (14).

The aim of this study was to investigate how introducing teledermatology affected the flow and management of skin tumours, both benign and malignant, from PCPs to dermatologists. The hypotheses were that introducing teledermatology would lead to: (*i)* increased dermato-scope use at PHCs; (*ii)* higher diagnostic accuracy among PCPs as a result; and (*iii)* shortened time intervals from initial investigation to melanoma excision.

## MATERIALS AND METHODS

Three independent PHCs in Östergötland County in south-east Sweden were selected as study units: 1 with a typical urban population, 1 with a mixed rural and suburban population, and 1 with a typical small-town population. Dermatoscopes were available in all 3 study units before the start of the study period. Teledermatology, the “store and forward” approach, was implemented at these healthcare centres starting in 2016, connecting them to the Dermatology Clinic at Linköping University Hospital. Before its introduction it was not possible to attach images to referrals. Teledermatological referrals were introduced as an option vs standard referrals with no incentive given in terms of faster acceptance or alternative management. If a PCP suspected a melanoma or deemed the situation higher priority a referral could be marked as “acute”, which is standard practice in Sweden in general. All patients with a skin tumour diagnosis matching those included in the search filter (see **Table I**) who registered 1 year before and 1 year after the implementation, were identified and selected through the electronic patient record system (Cambio Cosmic, Stockholm, Sweden).

**Table I:** Diagnoses by ICD-10 coding included in the study

D48.5 (unknown tumour of the skin)
L82 (seborrheic keratosis)
D22 (melanocytic nevus)
D23.9 (dermatofibroma)
I78.1 (non-neoplastic nevus – unspecified)
Q82.5 (congenital non-neoplastic nevus)
D18.0 (haemangioma)
L98.0 (pyogenic granuloma)
R23.8 (skin lesion – unspecified)
L57 (actinic keratosis)
L85 (skin horn + keratoacanthoma)
C44 (squamous cell carcinoma of the skin + basal cell carcinoma)
C43 (malignant melanoma)
D03 (melanoma in situ)
D04 (squamous cell carcinoma in situ)

Patient selection was followed by a retrospective patient record review. This resulted in a pre-teledermatology (PreT) cohort and a post-teledermatology (PostT) cohort. The patient record reviews were performed by 3 independent observers (CS, FME, and MBC), who reviewed records from 1 study unit each, with regard to a set of clinical variables, as displayed in **Table II**. Any uncertainties or disagreements regarding data extrapolation/interpretation were resolved by reaching consensus as a group to minimize observer bias. Based on the data extracted, diagnostic concordance between the initial lesion assessment made in primary healthcare and the diagnosis later set by the dermatologist, i.e., to what extent the diagnoses documented at the PCPs were in agreement with those of the dermatology clinic, was explored.

**Table II:** Data extracted from patients’ medical records

Number of lesions on the same patient Primary/suspected lesion diagnosis set by the primary care physician Final/suspected lesion diagnosis set by the dermatologist Histopathological diagnosis (if performed) Whether the final lesion diagnosis was based on histopathology or solely dermatologist assessment Age of patient at first visit to primary healthcare centre Sex of patient If it was fair to assume, based on the text in the medical journal, that a dermatoscope was used by the primary care physician If a referral was made and in the PostT cohort whether it was a teledermatological referral Number of days from primary healthcare visit to excision of a melanoma

Histopathological results were used as a reference standard when available, and dermatologist assessment was used as a reference standard when histopathology was not available but a referral had been made. When no referral was made, the primary care physician’s diagnosis was deemed correct. The correct diagnosis based on this classification will henceforth be known as the final diagnosis. Statistical analysis was conducted using IBM SPSS Statistics v.29.0.0.0 IBM Corp, Armonk, NY, USA). If a patient was lost to follow-up it was deemed an “incomplete evaluation” and therefore not included. If a patient presented with multiple lesions that were referred, they were counted as individual tumours but only as 1 referral. A χ^2^ test was used to compare the distributions of categorical variables between the cohorts, and Student’s two-sided paired *t*-test to compare means of continuous variables.

This is a retrospective cohort study. To avoid undesirable selection biases, no information or request to participate in the study was communicated. Ethical approval for the study was granted by the Swedish Ethical Review Authority, no. 2021-00193, prior to its initiation. For integrity protection, all data were pseudonymized during the process.

## RESULTS

**Fig. 2:**
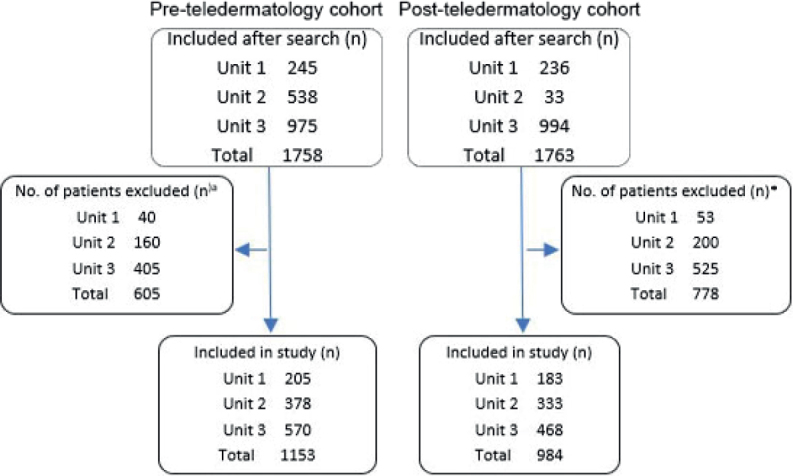
Inclusion/exclusion flowchart. ^a^Reasons for exclusion were the same in both cohorts: incorrect diagnosis, incomplete evaluation, visit took place outside of the study period, and the individual was already a patient at the dermatology clinic.

A total of 1,758 individuals were eligible for PreT after the initial search, 605 were excluded, and 1,153 were included in the study (see **Fig. 2**). In the PostT, 1,763 individuals were eligible after the initial search, 778 were excluded, and 984 were included, leading to a total of 2,137 study participants from both cohorts. Reasons for exclusion were the same in both cohorts:

- “Incorrect diagnosis”, most commonly the use of the ICD-10 code D48.5 (unknown tumour) on several other conditions, the most frequent being traumatic wounds as well as tumours not located on the skin.
- “Incomplete evaluation”, meaning that standard practice of skin lesion management was not possible due to external circumstances such as no dermatoscope present in nursing home or patient did not want referral/management of lesion because of age/comorbidities.
- “Visit took place outside of the study period”, meaning the diagnosis was documented during the study period but the first visit took place outside the study period.
- “The individual was already a patient at the dermatology clinic”, meaning the first visit took place before the study period.

### Comparison of general cohort data

**Table III** illustrates the distribution of clinical variables for the 2 cohorts. Mean patient age was 53.3 years in the PreT and 56.5 in PostT, with 516 men and 637 women in the former cohort, and 443 men and 541 women in the latter. Twenty-eight melanomas were found in the PreT and 16 were found in the PostT. The number of referred individuals totalled 321 in both cohorts. In the PostT group, 211 out of the 321, or 65.7%, were teledermatological referrals. In both cohorts the most common diagnosis in the age group 0–40 years was nevus and in the 40+ group seborrheic keratosis, as seen in **Table IV**.

**Table III:** Distribution of clinical variables in the PreT and PostT cohorts

	Individuals *n*	Diag-noses	MM	AK	SCC	BCC	Vasc	Lentigo	Nevus	SK	DF	Other	Referred individuals	Teledermato-logical referrals	MM days to manage-ment (mean)^a^	Dermato-scope used
PreT cohort																
Unit 1	205	243	8	34	7	18	10	3	67	79	5	12	82	0	63,4	33
Unit 2	378	465	7	41	14	42	12	2	124	156	13	53	103	0	60,3	250
Unit 3	570	689	12	67	12	32	31	2	308	197	7	21	136	0	46,4	298
Total	1,153	1,397	28	142	33	92	53	7	499	432	25	86	321	0		581
PostT cohort																
Unit 1	183	221	3	24	4	10	10	6	48	96	8	12	68	54	20.7	92
Unit 2	333	391	5	62	13	39	9	1	89	114	11	48	113	61	40,4	221
Unit 3	468	564	8	57	13	45	18	3	202	190	8	20	140	96	66,4	313
Total	984	1,176	16	143	30	94	37	10	339	400	27	80	321	211		626

a Counted from day of referral.

Vasc: vascular lesion; SK: seborrheic keratosis; DF: dermatofibroma; MM: malignant melanoma; AK: actinic keratosis; SCC: squamous cell carcinoma; BCC: basal cell carcinoma.

**Table IV:** Tumour distribution in age groups

Diagnosis	MM	SCC	BCC	Nevus	SK
Patient aged < 41 PreT/PostT	2/1	1/1	0/1	305/196	31/34
Patient aged > 40 PreT/PostT	26/15	32/30	92/93	194/143	401/366

### Dermatoscope use

Frequency of dermatoscope use by PCP before and after implementation is indicated in **Table V**. Dermatoscope use significantly increased for Unit 1 and Unit 3 (*p* < 0.001) but not for Unit 2 (*p* = 0.410), calculated using a χ^2^ test.

**Table V:** Frequency of dermatoscope use by primary care physician before and after implementation

PHC	Dermatoscope use PreT	Dermatoscope use PostT	*p*-value
Unit 1	33/243 = 13.6%	92/221 = 41.6%	< 0.001
Unit 2	250/465 = 53.8%	221/391 = 56.5%	0.410
Unit 3	298/689 = 43.3%	313/564 = 55.5%	< 0.001

### Concordance between clinicians

The concordance between initial lesion diagnoses suspected by the PCPs, diagnoses made by dermatologist assessment, and diagnoses stated by histopathology in the PostT cohort compared with the PreT cohort can be seen in **Table VI**. Unit 1 showed an increase in the proportion of final diagnoses made solely by the PCP and a decrease in the number made at the dermatology clinic. Conversely, Units 2 and 3 showed a decrease in the proportion of final diagnoses made by PCPs and an increase in the proportion of final diagnoses made at the dermatology clinic. Units 1 and 2 showed an increase in the proportion of coherent diagnoses between clinics, while Unit 3 showed a decrease.

**Table VI:** Who made the final diagnosis and diagnostic concordance

Primary healthcare centre	Diagnosis suspected by primary care physician concordant with reference diagnosis *n* (%)	Diagnosis made by dermatologist concordant with reference diagnosis *n* (%)	Same diagnosis from both clinics *n* (%)	Total number of diagnoses^a^
Unit 1				
PreT	159 (65.4)	38 (15.6)	46 (18.9)	243
PostT	149 (67.4)	23 (10.4)	49 (22.2)	221
Unit 2				
PreT	338 (72.7)	68 (14.6)	59 (12.7)	465
PostT	252 (64.5)	63 (16.1)	76 (19.4)	391
Unit 3				
PreT	547 (79.4)	95 (13.8)	47 (6.8)	689
PostT	409 (72.5)	120 (21.3)	35 (6.2)	564

a No diagnosis exists in more than one field in the table.

### Melanoma referral time

**Fig. 3:**
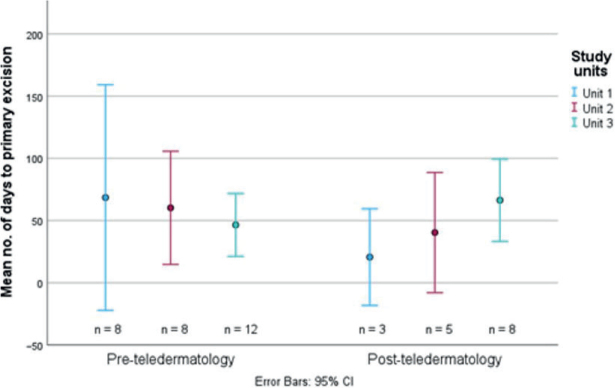
Distribution of days from referral to primary excision of detected malignant melanomas in the 2 study cohorts, illustrated as mean number of days, with 95% confidence intervals (CI).

Distributions between the 2 cohorts in days from referral to management/excision of lesions when the histopathological diagnosis was malignant melanoma are illustrated in **Fig. 3**. The mean number of days until excision of a melanoma was, per unit, 1–3: 68.5, 60.3, and 46.5 in the PreT, and 20.7, 40.4, and 66.4 in the PostT, respectively, resulting in a PreT mean of 56.7 days and a PostT mean of 49.7 days (*p* = 0.705, two-sided paired *t*-test).

## DISCUSSION

Introducing a new diagnostic tool can take time to incorporate into practice and use in the clinical setting. Our results show that almost 66% of referrals were teledermatological in the year following its introduction, showing an adaptation to the new referral system despite lack of added incentive to use it, making it easier to compare the two cohorts in a favourable way for this study. There is very little data from previous studies with which to compare this implementation fraction, and it is also probable – given that the healthcare system is a huge apparatus that tends to “move slowly” – that if a follow-up study was to be made a few years later, that number would most likely be higher. The introduction of teledermatology with dermoscopic images as an obligatory part of a referral for a suspicious lesion was chosen from the beginning to increase diagnostic ability of the dermatologist receiving the referral. Bouton *et al*. also showed that transmission of only macroscopic photographs of suspected melanoma lesions did not lead to significant improvement of the patient care pathway and did not improve patient compliance (15). There are also very few referrals going from PHC to private dermatologists so there is no monetary gain in accepting referrals, making these results less affected by that possible confounder.

The use of dermoscopy by the examining PCP showed a clear increase in the post-teledermatology group in 2 of the 3 primary healthcare centres (units 1 and 3). Unit 2 had two physicians with a special interest in dermatology and dermoscopy employed at the time who were already proficient in their use of dermoscopy, which could explain this between-group deviance. These findings are supported by Rosendahl et al. (16), who discussed these advantages of subspecialized general practitioners in skin cancer in 2012 showing fewer unnecessary excisions and a higher use of dermoscopy. Although not statistically significant, there was a slight percentage increase in the post-teledermatology cohort in this unit as well. Awareness of dermoscopy and courses directed at PCPs had not yet been adopted on a broader scale in the county of Östergötland, but was only occasionally used, primarily by a limited number of interested physicians, so it is less likely that it would have affected the overall use of dermoscopy. This increase in dermoscopy use following the implementation of teledermatology appears to be a novel finding, based on the lack of publications presenting similar findings or studies.

Looking at diagnostic concordance there was a trend towards a more concordant view between the PHCs and dermatology clinic in the PostT cohort, as seen in Table VI. This might be an indication of increased diagnostic ability, probably at least partly due to increased dermatoscope use at the PHCs. The concordance seen in this study is not as high as in some previous studies, although exact comparisons are hard to make. Previous studies were based on the full spectrum of teledermatological referrals and not just on skin neoplasms, and most compared a dermatologist undertaking a teledermatology assessment with a face-to-face visit (6, 17). We also used histopathology as the reference standard when available, negatively affecting concordance.

Although a few previous studies have shown a reduction in melanoma management time (10), with a population of 2,100 patients, the diagnosis of melanoma is not abundant, which makes it more difficult to draw conclusions from the results. We only looked at lesions which received the histopathological diagnosis of melanoma and not lesions that were suspected to be melanomas at first visit but later received a different clinical or histopathological diagnosis. Therefore, these numbers included a few statistical outliers due to awaiting biopsy results as well as some short-term monitoring of suspected lesions. A follow-up study looking more in-depth at melanomas and melanoma management a few years after the implementation of teledermatology would most likely give a more accurate representation of the real-world scenario.

The study design allows for a natural consecutive selection of patients for a relatively long inclusion period of 1 year for each cohort. All 3 observers are medical doctors and are very familiar with this patient cohort, and Östergötland County has been shown to be a good statistical representative for the country as a whole (14). This should contribute to high internal validity and external validity as well as to high reliability and generalizability for primary healthcare in Sweden in general, and for the south-east region in particular. Internal validity is lowest when it comes to the use of dermoscopy by the PCP, because it is based on the visit documentation made by the physicians themselves, as done in previous studies (18). However, thorough measures to reach consensus were taken to minimize observer bias. A strained healthcare system suffering from short patient visits could have resulted in the exclusion of this information in patient journals because of time constraints but, if so, it is reasonable to assume it would have affected both cohorts evenly. A limitation in the study material is the lack of information on the level of clinical experience of the PCPs assessing the lesions in the material, both with regard to using a dermatoscope and to educational level, a fact that might affect interpretation and generalization of the results.

Considering the observed increase in the use of dermoscopy as well as the use of teledermatological referrals after such a short time (one year following its implementation), it is likely to assume that the PCPs’ diagnostic accuracy, dermatoscope use, and use as well as quality of teledermatological referrals will continue to increase over time, especially with more courses in dermoscopy present. Marra et al. also showed this, i.e., that PCPs who followed a skin cancer training programme have better diagnostic skills and quality of referrals than untrained colleagues and this in turn would most likely result in fewer unnecessary referrals (19, 20). A longer time gap between the 2 cohorts could have possibly highlighted potential improvements/differences even more. The material also did not allow for calculation of diagnostic accuracy measures, as we lack prospective information on lesions not referred that might later prove to be skin cancer.

Another way to increase accessibility, in the near future, to secondary care for skin cancer patients in need of more rapid management by decreasing unnecessary workload for dermatologists and pathologists and reducing the effect variance in diagnostic skill among PCPs, might be through artificial intelligence-based decision support as shown recently by Papachristou et al. (21). This, combined with previously mentioned efforts, is likely to strengthen skin cancer care in the future despite increasing patient numbers.

In conclusion, teledermatology has, in several studies, proved to be a valuable tool when diagnosing benign, cancerous, and pre-cancerous skin lesions and has also improved communications between PHC and dermatology clinics. This is also supported by the results of our study, indicating a higher level of concordance between the diagnostic assessment made by PCPs and those made by dermatologists after implementation of teledermatology, as well as an increase in the use of dermoscopy in the primary healthcare setting. Potentially wider use of teledermatology may shorten the overall time to diagnosis of melanomas, as well as referral time and number of days to excision, and may contribute to more efficient management of this patient group.
